# Deficiency of DEK proto‐oncogene alleviates allergic rhinitis by inhibiting RhoA/Ezrin‐mediated mitochondrial fission

**DOI:** 10.1002/ame2.12523

**Published:** 2024-12-12

**Authors:** Longzhu Dai, Yongde Jin, Jingmei Chai, Jianing Yang, Jiangang Wang, Mu Chen, Liangchang Li, Chongyang Wang, Guanghai Yan

**Affiliations:** ^1^ Jilin Key Laboratory for Immune and Targeting Research on Common Allergic Diseases Yanbian University Yanji P. R. China; ^2^ Department of Anatomy, Histology and Embryology Yanbian University Medical College Yanji P. R. China; ^3^ Department of Otorhinolaryngology Affiliated Hospital of Yanbian University Yanji P. R. China; ^4^ Department of Traditional Chinese Medicine Yanbian University Medicine College Yanji P. R. China

**Keywords:** allergic rhinitis, DEK, Ezrin, house dust mite, mitochondria

## Abstract

**Background:**

Allergic rhinitis (AR) is a kind of immune disease mediated by IgE. We are intrigued by the potential role of DEK proto‐oncogene (DEK) in inflammation‐related diseases. We investigated the effects and mechanisms of DEK in treating AR, aiming to identify potential new treatment targets for AR.

**Methods:**

The AR mouse model was induced by house dust mite (HDM) (1 mg/mL). HNEpCs stimulated by HDM (1 mg/mL) were pretreated for 24 h with or without DEK lentivirus. The effect of DEK knockout or knockdown on AR was evaluated in vitro and in vivo using western blotting, ELISA, flow cytometry, real‐time quantitative PCR, immunohistochemistry, HE staining, PAS staining, Diff staining, and immunofluorescence.

**Results:**

After DEK knockdown, the inflammatory response of AR mice was reduced. In addition, DEK deletion mitigated nasal tissue damage and mitochondrial division. Our further studies showed that DEK deletion or inhibition led to the down‐regulation of RhoA activity and decreased phosphorylation of Ezrin and Drp1 proteins, and inhibited mitochondrial division. Overall, DEK deficiency mitigated AR by down‐regulating RhoA/Ezrin/Drp1 pathway activity.

**Conclusion:**

DEK alleviates AR through RhoA/Ezrin/Drp1 signaling pathway, which provides a new perspective for developing improved therapies and understanding the pathogenesis of AR.

## Graphical abstract

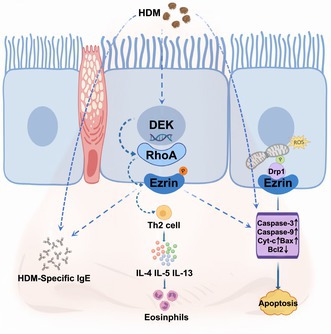

## 1 INTRODUCTION

Allergic rhinitis (AR) is an upper respiratory disease mediated by immunoglobulin E (IgE), which triggers an immune response upon exposure to external allergens.^1^ It is currently a global health concern. The incidence of AR, asthma, and other chronic respiratory diseases has been increasing annually, possibly due to air pollution and climate change in recent years.^2^ In the past 6 years, the standard prevalence of AR among adults in China has increased by 6.5%.^3^ Currently, several commonly used clinical treatments for AR, including hormones, antibiotics, and antihistamines, remain limited.^4^ The primary reason is the unclear pathogenesis of AR, making it essential to explore its pathogenesis and treatment targets.

DEK proto‐oncogene (DEK), an oncogene, is highly expressed in most human tissue cells and is overexpressed in developing cancers. It influences various cellular processes, such as cell proliferation, differentiation, and aging.^5^ Mor Vakknin et al.^6^ demonstrated that DEK was a nuclear phosphoprotein and an autoantigen, and its antibodies, closely related to T‐cell apoptosis, were also present in diseases related to abnormal immunity. Studies on DEK and asthma have revealed the involvement of DEK in regulating mitochondrial structure and function.^7^, ^8^ Overexpression of DEK leads to mitochondrial dysfunction and structural damage, whereas inhibition of DEK can reduce airway inflammation and alleviate respiratory diseases.^9^ Considering the unclear mechanism of DEK's involvement in airway inflammation, we further studied the relationship between DEK and AR, one of the respiratory diseases.

RhoA is a crucial member of the Rho protein, a cytoplasmic protein that belongs to the subfamily of the small G protein superfamily. RhoA can bind with chemokines, growth factors, and cytokines, leading to its activation. On one hand, it interacts with innate immune cells, which can be activated by pathogens and danger signals and can migrate to sites of inflammation. On the other hand, it also presents pathogen‐derived antigens and induces the activation of adaptive T cells through immune synapses, contributing to the formation of antigen‐specific T cells.^8^ Disruption of RhoA reduces allergic airway inflammation by inhibiting T‐cell activation and TH2 differentiation, but it has no effect on TH1 cells.^10^ Crucially, RhoA can prevent the development of allergic airway inflammation.^10^ Consequently, we speculate that DEK may be involved in the occurrence of AR by regulating RhoA.

Ezrin is the first protein identified in the ERM protein family, implicated in cell proliferation, adhesion, migration, membrane protein modulation, and vesicle transport. Additionally, Ezrin functions as an upstream effector of RhoA and is involved in the occurrence of neuritis.^11^ It is involved in respiratory diseases,^12^ affecting airway cell function, and can mediate the occurrence of asthma^13^ through bronchial epithelial repair, T lymphocyte regulation, and airway smooth muscle contraction.^14^ The interaction between Ezrin and RhoA is well known,^15^ and multiple studies have confirmed the impact of their related signaling pathways on different diseases.^16^ However, there is currently no report on their association with AR.

In the present study, we demonstrate that house dust mite (HDM) treatment induces an upregulation of DEK expression, activation of the RhoA/Ezrin signaling pathway, and an increase in mitochondrial fission, both in vitro and in vivo. Subsequent investigations reveal that after the knockout of DEK, the HDM‐induced inflammatory response and activation of the RhoA/Ezrin signaling pathway are inhibited. Mechanistically, we provide evidence that DEK knockout modulates mitochondrial fission by regulating HDM‐induced RhoA/Ezrin signaling pathway activation. Notably, the inhibition of DEK may mitigate the inflammatory response and thereby ameliorate AR. Consequently, the DEK^−/−^/RhoA/Ezrin signaling pathway holds promise for advancing the understanding of AR pathogenesis and developing novel AR therapeutics.

## 2 MATERIALS AND METHODS

### 2.1 Mice

Male C57Bl/6 mice (6–8 weeks) were purchased from Yanbian University Health Science Center (Yanji, China), and DEK knockout (DEK^−/−^) mice were obtained from Cyagen (Suzhou, China). They were housed in specific pathogen‐free conditions at a temperature of 22 ± 2°C and a relative humidity of 50%–60%. All animals were used in accordance with the Laboratory Animal Use Permit of Jilin Provincial Department of Science and Technology (SYXK (JI) 2020–0009). The experiment procedures were approved by the Laboratory Animal Ethics Committee Yanbian University (number: YD20231027002).

### 2.2 HDM‐induced AR model

Wild‐type mice and DEK^−/−^ mice were randomly divided into the control group, the HDM‐AR model group, the DEK^−/−^ group, and the DEK^−/−^ + HDM group, with 20 mice in each group. Over a period of 0–26 days, mice were subjected to daily nasal injections of 20 μL of HDM (1 mg/mL, Greer Laboratories, Lenovo, NC, USA) extract or 20 μL of phosphate buffered saline (PBS). They were killed on the 27th day, 24 h after the last nasal treatment^17^ (Figure 2A). After the last stimulation, behavioral observation was performed on these mice, and the success of the AR model was initially judged by the number of sneezing and rubbing.

### 2.3 Real‐time quantitative polymerase chain reaction

To detect the change in DEK expression at the level of nucleic acid, RNA was extracted from mouse nasal mucosal tissues and cells treated with HDM using a Fastking Total RNA extraction kit (Tiangen, DP451, Beijing, China). The complementary DNA (cDNA) was synthesized using a FastKing RT kit (Tiangen, KR118‐02). Real‐time quantitative polymerase chain reaction (RT‐qPCR) was conducted using a SYBR green RT‐qPCR kit (Tiangen, KR123) on the Azure cielo 6 system (Azure, Dublin, CA, USA). The PCR procedure was carried out with an initial denaturation at 95°C for 15 min, followed by 40 cycles at 94°C for 10 s, 55°C for 20 s, and 72°C for 20 s. The DEK and glyceraldehyde‐3‐phosphate dehydrogenase (GAPDH) primers were obtained from Sangon Biotech (Shanghai, China), with their details provided in Table 1.

**TABLE 1:** Primer sequences.

Name	Sequence
mmu‐DEK forward	5′‐AACGTGGGTCAGTTCAGTGGC‐3′
mmu‐DEK reverse	5′‐TTCGCTGTTCACGCCTGACCT‐3′
β‐actin forward	5′‐CAGCCTTCCTTCTTGGGTATG‐3′
β‐actin reverse	5′‐GGCATAGAGGTCTTTACGGATG‐3′

### 2.4 Immunofluorescence

To reveal the protein expression levels in vivo and vitro, the cells or nasal tissue sections were fixed with 4% paraformaldehyde at room temperature for 10 min and then washed with PBS for 5 min thrice. The cell membrane was permeated with 0.2% Triton X‐100 at room temperature for 1 h and then washed with PBS thrice for 5 min each. Following this, the samples were blocked with 5% bovine serum albumin (BSA) for 1 h and washed with PBS thrice for 5 min each. Then, they were subjected to overnight incubation with the primary antibodies of anti‐phospho‐Drp1 (Ser616) (p‐Drp1^(616)^) (#AF8470, Affinity Biosciences, Cincinnati, OH, USA), anti‐Drp1 (611 738, BD, China), anti‐Tom20 (ab56783, ab186735, Abcam, USA), anti‐Ezrin (ab4069, Abcam), and anti‐phospho‐Ezrin (T567) (p‐Ezrin) (ab47293, Abcam). Subsequently, the corresponding secondary antibodies, Alexa Fluor 488 goat anti‐rabbit (A11001, Thermo Fisher Scientific, Waltham, MA, USA) and Goat Anti‐Mouse IgG H&L (Cy3®) (ab97035, Abcam), were incubated at room temperature for 2 h, followed by washing with PBS thrice for 5 min each. Finally, the cells were treated with an antifluorescence quenching agent, including DAPI (4 ', 6‐diamidino‐2‐phenylindole), a DNA dye that fluoresces blue, for 2 h and then photographed using a Cytoation5 fluorescence microscope (BioTek, Inc., Winooski, VT, USA).

### 2.5 Western blotting

Western blotting (WB) analysis was conducted to assess the protein expression levels. Nasal tissue and cell samples were homogenized and then lysed using RIPA buffer (Radio Immunoprecipitation Assay Lysis Buffer) (Beyotime, Shanghai, China). The concentration of all extracted proteins was determined using a bicinchoninic acid kit (P0010S, Beyotime). Subsequently, 20 μg of the proteins was separated u sodium dodecyl sulfate‐polyacrylamide gel electrophoresis (SDS‐PAGE). The expression level of the target protein was determined after electrophoretic transfer into polyvinylidene fluoride (PVDF) membranes (Millipore, Billerica, MA, USA), followed by incubation with the primary antibodies at 4°C overnight and the secondary antibody at 37°C for 1 h. The primary antibodies included anti‐Ezrin, anti‐β‐actin (8H10D10) (#3700s, Cell Signaling Technology, CST, Danvers, MA, USA), anti‐p‐Ezrin, anti‐DEK (#DF12097, Affinity), anti‐ROCK1 (ab134181, Abcam), anti‐Cytochrome C (cyt‐c) (ab133504, Abcam), anti‐Drp1 (ab184247, Abcam), anti‐Bcl‐2 (ab194583, Abcam), anti‐cleaved‐caspase‐3 (Asp175) (5A1E) (#9664, CST), anti‐cleaved‐caspase‐9 (Asp315) (D8I9E) (#20750S, CST), anti‐p‐Drp1^(616)^, anti‐Phospho‐Drp1 (Ser637) (p‐Drp1^(637)^) (#DF2980, Affinity), anti‐Bax (#19684, Abclonal, WuHan, China), and anti‐RhoA (ab187027, Abcam). The secondary antibodies were anti‐rabbit IgG (H + L) (DyLight800 4X PEG Conjugate) (#5151, CST) and anti‐mouse IgG (H + L) (DyLight800 4X PEG Conjugate) (#5257, CST). Finally, the proteins were analyzed using a Luminescent Image Analyzer (76120280, GE Healthcare Bio‐Sciences AB, Japan).

### 2.6 Histological analysis and immunohistochemistry

To determine whether allergic inflammation occurs in mouse nasal tissues and the expression changes in related pathway proteins between different groups, the following three kinds of staining were performed. The nasal tissue was fixed in 4% paraformaldehyde for 3 days and decalcified in ethylenediaminetetraacetic acid (EDTA) (pH = 7.2) (E1171, Solarbio, China) for 14 days. Subsequently, the tissue was embedded in paraffin and cut into 4‐μm sections, which were then stained with hematoxylin–eosin (H&E) for eosinophils and with periodic acid‐Schiff for goblet cells. Immunohistochemical staining was conducted for DEK, p‐Ezrin, and cyt‐c. The sections were incubated overnight at 4°C with rabbit anti‐DEK, rabbit anti‐p‐Ezrin, and rabbit anti‐cyt‐c antibodies. Subsequently, they were incubated with secondary antibodies using the immunohistochemistry kit (PV9000, Zhongshan Jinqiao, Beijing, China). Ultimately, the nasal tissue sections were analyzed with a Slide Scanning System (SQS‐40P, TEKSQRAY, Shenzhen, China).

### 2.7 Flow cytometry

To observe changes in eosinophils, nasal lavage fluid (NALF) obtained from killed mice was quickly centrifuged to obtain cell precipitates. The cells were then stained with eBioscience Fixable Viability Dye eFluor 780 (65‐0865‐14, Thermo Fisher Scientific), gently resuspended by pipetting and incubated at 4°C in the dark for 30 min. Pre‐cooled PBS was used for washing, followed by centrifugation and discarding of the supernatant. The cells were subsequently stained with an APC‐conjugated CD45.2 antibody (#109814, Biolegend, CA, USA), Percp Cy5.5‐conjugated CD3e antibody (45‐0031‐82, Thermo Fisher Scientific), and PE‐conjugated SiglecF antibody (#155506, Biolegend) at 4°C in the dark for 30 min. Again, pre‐cooled PBS was used for washing, followed by centrifugation and discarding of the supernatant. Then, 300 μL of pre‐cooled PBS was added, and the sample was analyzed using Cytoflex S (Beckman Coulter, Inc., Brea, CA, USA). Dimers and agglomerates were separated by setting up FSC‐A versus FSC‐H and SSC‐A versus SSC‐H, and the analysis was conducted using Cytoexpert 2.4 software with the CytoFLEX (A00‐1‐1102, Beckman Couter Biotechnology, Suzhou, China).

### 2.8 Enzyme‐linked immunosorbent assay

The levels of HDM‐specific IgE in the nasal mucosa, as well as interleukin 4 (IL‐4), IL‐5, and IL‐13 in the serum, were measured using ELISA kits (Mlbio, Shanghai, China). It's to determine the severity of allergic response in mice.

### 2.9 Cell treatment and transfection

The primary human nasal epithelial cells (HNEpCs) were obtained from Saibaikang Guangzhou Genio Biotechnology Co., Ltd. (Guangzhou, China). They were cultured in a 37°C, 5% CO_2_ incubator in iCell primary epithelial cell culture medium (PriMed‐iCell‐001) supplemented with 2% fetal bovine serum (SAIBAIKANG, Shanghai, China). To suppress DEK and Ezrin expression in HNEpCs, we transfected HNEpCs with small interfering RNA (siRNA) targeting DEK (20 μmol/L, RIBOBIO, Guangzhou, China) and siRNA targeting Ezrin (20 μmol/L, RIBOBIO) for 48 h using Lipofectamine 3000 (13 778 030, Thermo Fisher Scientific). During this time, HDM (10 μg/mL, Greer Laboratories) was added and incubated for 24 h. For stimulation, rmDEK was administered at 0.1, 1, and 10 μg/mL for 24 h.

### 2.10 Assessment of reactive oxygen species generation

To detect the levels of reactive oxygen species (ROS) for determining the degree of cell damage, DCFH‐DA (S0033S, Beyotime) was added and incubated at 37°C for 30 min. For the measurement of mitochondrial ROS, cells were incubated with MitoROS dye (M36008, Thermo Fisher Scientific) at 37°C for 30 min. The data were collected using Cytation5.

### 2.11 Mitochondrial membrane potential and TUNEL assay

For the detection of mitochondrial membrane potential, the HNEpCs were treated at room temperature with a JC‐1 reagent kit (C2006, Beyotime) and then observed using Cytoation5. Furthermore, we used the one‐step TUNEL (TDT‐mediated Dutpnick‐endlabeling) cell apoptosis detection kit (C1090, Beyotime) to detect cell apoptosis. When genomic DNA is broken, the exposed 3 '‐OH can be labeled with fluorescein (FITC) catalyzed by terminal deoxynucleotide transferase and thus can be detected by fluorescence microscopy or flow cytometry. The paraffin tissue sections were deparaffinized and incubated with DNase‐free protease K (20 mg/mL, ST532, Beyotime) for 30 min. After being washed with PBS thrice, each time for 3 min, the sections and cells were incubated with 50 μL of the TUNEL detection solution at 37°C for 1 h. Finally, the samples were observed with Cytoation5 after fluorescence quenching.

### 2.12 Statistical analysis

The results were expressed as mean ± standard deviation. Intergroup comparison was made using one‐way analysis of variance (ANOVA) followed by Duncan's multiple‐range tests. A *p*‐value of <0.05 was deemed to be statistically significant. Data analysis was performed using SPSS 17.0 and GraphPad Prism 7.0.

## 3 RESULTS

### 3.1 Expression of DEK is upregulated in the nasal mucosa of HDM‐induced AR mice

**FIGURE 1:**
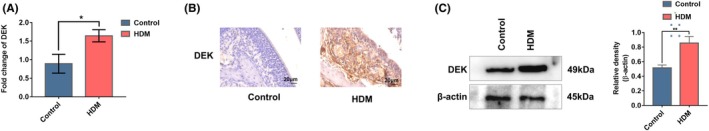
House dust mite (HDM) can induce DEK overexpression. (A) The messenger RNA (mRNA) levels of DEK in mice were detected using real‐time quantitative polymerase chain reaction (RT‐qPCR). (B) Immunohistochemistry was used to detect the DEK expression in the nasal tissues of mice. Relative protein levels were determined by calculating the ratio of DEK to β‐Actin density, and the results are expressed as mean ± standard deviation. (C) DEK protein levels in the nasal mucosa of mice with or without allergic rhinitis (AR) were determined using Western blotting (WB). Scale bar = 20 μm; magnification was 200×; *p < 0.05, **p < 0.01.

Our previous study demonstrated that DEK overexpression resulted in significant mitochondrial dysfunction in asthma, characterized by upregulation of Drp1, downregulation of p‐Drp1^(637)^ and mitochondrial fusion protein 2 (MFN2), loss of mitochondrial membrane potential, excessive generation of mitochondrial ROS, and mitochondrial incompleteness.^18^ However, according to the literature review, AR is often comorbid with asthma.^19^ Therefore, we further explored the relationship between DEK and AR. We first examined the messenger RNA (mRNA) expression of DEK, which significantly increased in the nasal mucosa of HDM‐induced AR mice compared to that of the control mice (Figure 1A). Moreover, we conducted an immunohistochemistry analysis to demonstrate the presence of DEK in nasal tissues of AR‐induced mice (Figure 1B). Additionally, WB analysis showed strong expression of DEK in the nasal mucosa of AR mice but not in control mice (Figure 1C). These results showed that DEK expression was upregulated in nasal tissues with AR, indicating that DEK might play a role in the development of AR.

### 3.2 DEK knockout alleviates the development of HDM‐induced AR

**FIGURE 2:**
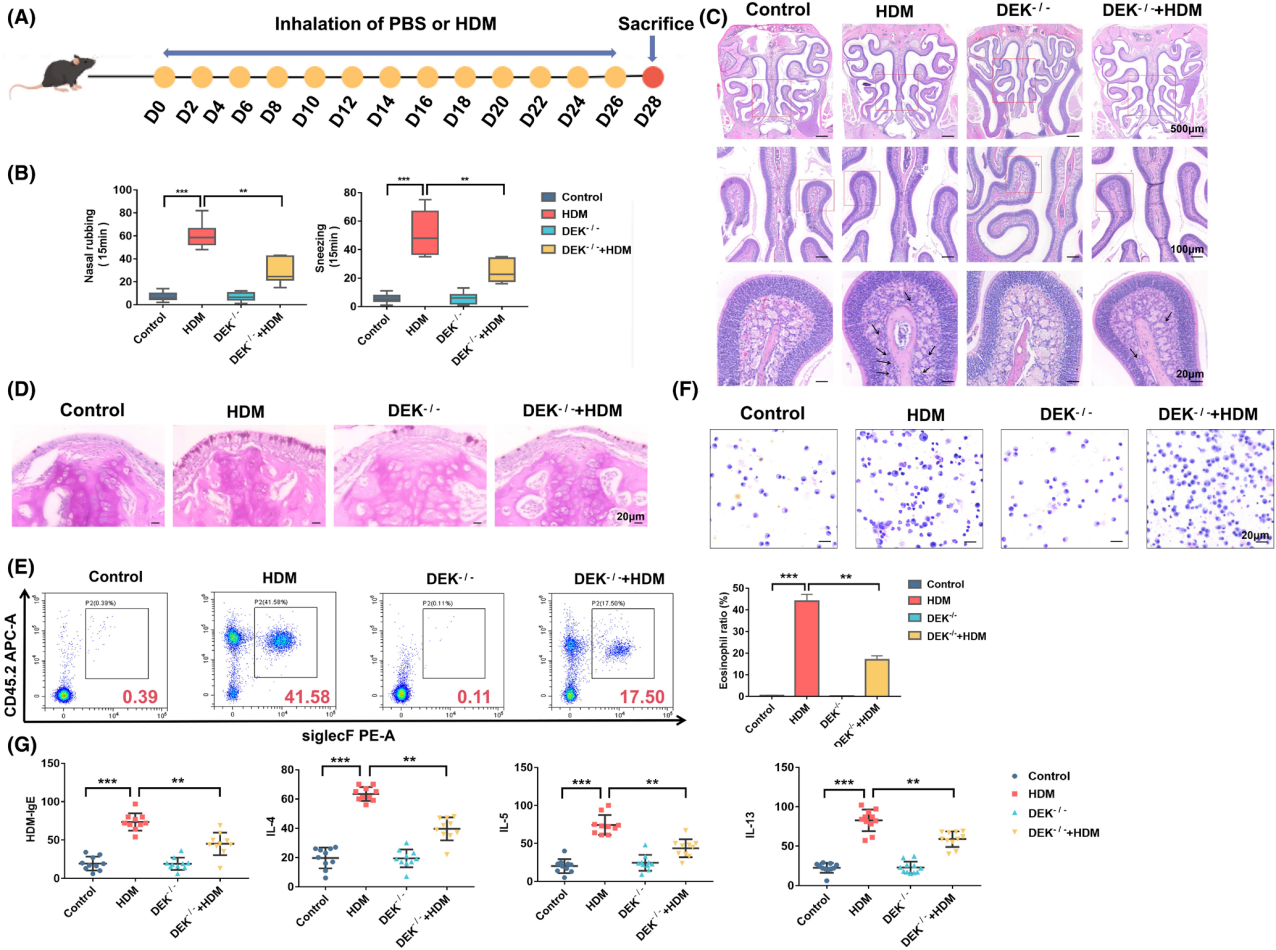
DEK knockout alleviates house dust mite (HDM)‐induced inflammatory response in allergic rhinitis (AR) mice. (A) Experimental protocol for HDM and phosphate buffered saline (PBS) treatment. (B) Frequency of sneezing and nasal rubbing in mice. (C, D) Images of hematoxylin–eosin (H&E)‐stained and periodic acid‐Schiff–stained nasal tissue sections. (E) Flow cytometry analysis of eosinophils in the nasal lavage fluid. (F) Expression of eosinophils by Diff staining. (G) Expression of HDM‐IgE, IL‐4, IL‐5 and IL‐13 by ELASA. Magnification was 40×, 100×, and 200×; **p < 0.01 and ***p < 0.001.

Nasal rubbing and sneezing are the primary symptoms of AR.^20^ We then recorded the frequencies of nasal rubbing and sneezing for 15 min following the final intranasal HDM treatment on day 27 (Figure 2A). We found that the number of sneezing and rubbing in HDM‐induced group increased significantly, whereas the number of nasal sneezing and rubbing in the DEK^−^/^−^ + HDM‐induced group decreased significantly than that of the HDM‐induced group (Figure 2B). Eosinophils are extremely important cells in immune responses and allergic diseases. Therefore, we detected the levels of eosinophils in the nasal tissue using HE staining. The results showed that a large number of eosinophils appeared in the paraffin sections of wild‐type mice induced by HDM. Combined with the results of behavioral characteristics of mice, we successfully established the AR model. Compared with the HDM‐induced group, the level of eosinophils in the nasal tissue of DEK^−^/^−^ + HDM‐induced group was significantly reduced (Figure 2C). Similarly, periodic acid‐Schiff staining showed that there was a significant increase in goblet cells in AR (Figure 2D). In addition, because the NALF can only remove a very small number of cells, we collected cells from the bronchoalveolar lavage fluid (BALF), which is also the respiratory tract. The proportion of eosinophils in the BALF of AR mice increased, whereas it was significantly reduced in the DEK^−^/^−^ + HDM‐induced group (Figure 2E,F). Because the occurrence of AR affects the changes in some TH2 cytokines, such as IL‐4, IL‐5, and IL‐13, which are responsible for the IgE production of B cells and the stimulation and recruitment of eosinophil leading to eosinophil proliferation, we further investigated whether DEK^−^/^−^can lead to the reduction of these cytokines. HDM treatment increased the production of IL‐4, IL‐5, IL‐13, and IgE in NALF, and the levels significantly decreased in mice after DEK knockout (Figure 2G). These findings suggest a significant alleviation of the inflammatory response in AR mice due to DEK knockout.

### 3.3 DEK knockout inhibits nasal tissue damage and reduces mitochondrial fission in AR mice

**FIGURE 3:**
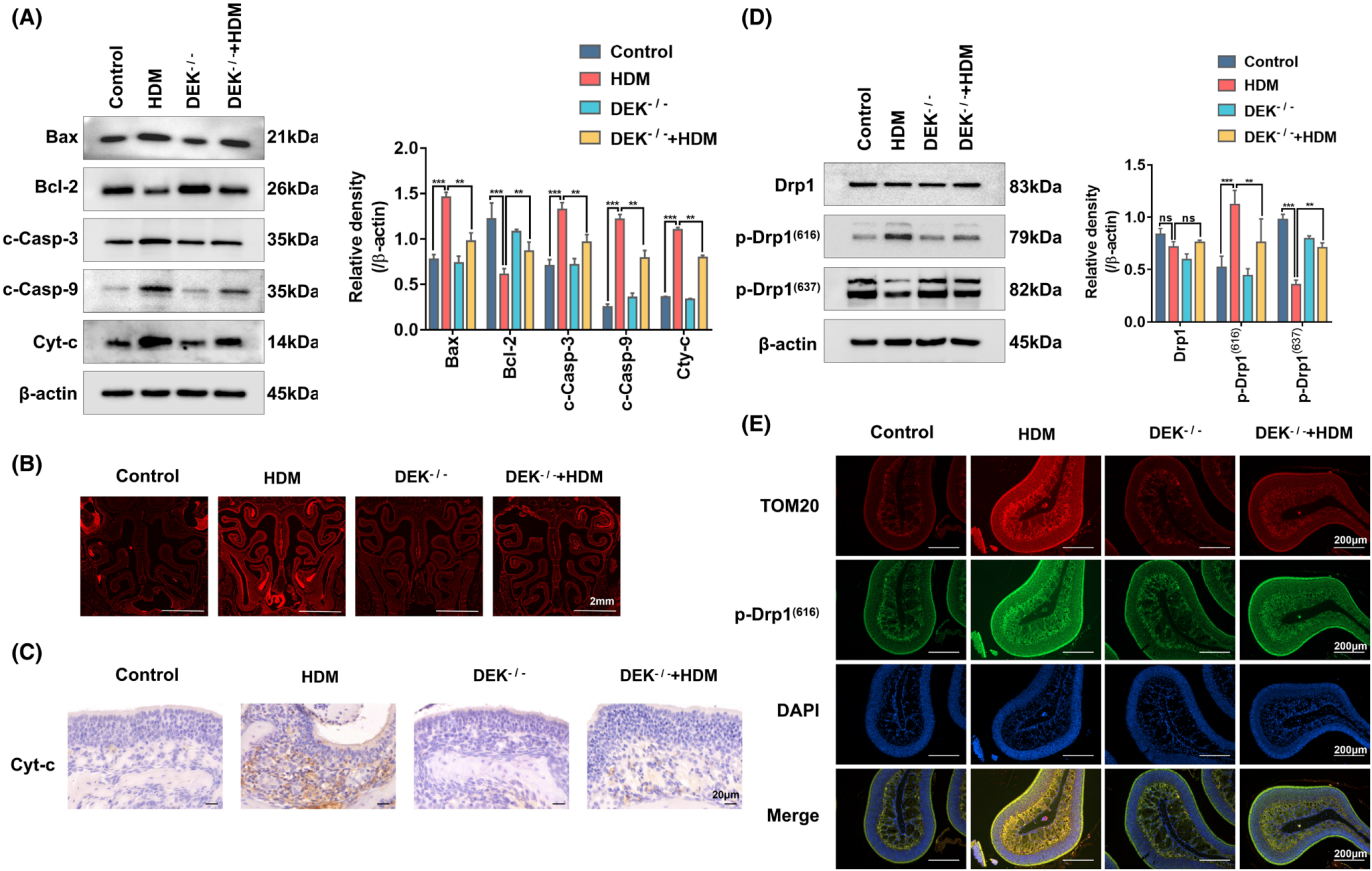
DEK knockout alleviates nasal tissue damage and reduces mitochondrial fission in AR mice. (A) Western blotting (WB) analysis of apoptosis‐related protein levels in the nasal mucosa of mice. (B) The apoptosis in nasal tissue sections of mice was examined through a TUNEL assay. (C) Immunofluorescence staining was used to determine the expression of cyt‐c in the nasal sections of mice. (D) WB analysis of Drp1, p‐Drp1(616), and p‐Drp1(637) protein levels in the nasal tissue mucosa of mice. (E) Tom20 assay was performed to determine the expression and cellular localization of p‐Drp1(616) in the nasal sections of mice. Relative protein levels were determined by dividing the density of the target proteins by that of β‐Actin, and the results are presented as mean ± standard deviation. Magnification was 200×; ns, not significant; **p < 0.01 and ***p < 0.001.

During tissue repair, certain mechanisms opt for apoptosis as a means of repair, leading to a significant increase in apoptotic activity.^21^ Based on our initial findings,^9^ we hypothesized that DEK knockout could inhibit the development of AR, prompting further investigation into its potential to mitigate nasal tissue damage. Initially, the expression levels of apoptotic proteins, such as cyt‐c, cleaved‐caspase‐3, cleaved‐caspase‐9, Bax, and Bcl‐2, in each group were assessed using WB (Figure 3A). Furthermore, the level of apoptosis in tissue sections was examined using TUNEL assay and immunohistochemistry (Figure 3B,C). Our results indicated significant upregulation of apoptosis in AR model mice compared to control mice, whereas the apoptosis level in AR mice sensitized with DEK knockout was somewhat alleviated.

Apoptotic pathways are categorized into endogenous mitochondrial, endogenous endoplasmic reticulum, and exogenous death receptor pathways.^22^ This study investigated the effect of DEK on mitochondrial fission and apoptosis. The results revealed no significant difference in total Drp1 expression. However, HDM stimulation augmented the phosphorylation of Ser616 and diminished the phosphorylation of Ser637 in Drp1, signifying its activation. Notably, the DEK knockout reversed this alteration (Figure 3D). Furthermore, the assessment of Tom20 and p‐Drp^(616)^ co‐localization in HNEpCs revealed a substantial increase in their co‐localization in AR mouse nasal tissue. Conversely, this co‐localization was partially mitigated in AR mice with DEK knockout, suggesting a reversal of mitochondrial fission (Figure 3E). Altogether, these data suggest that downregulating DEK expression may mitigate tissue damage and cell apoptosis in AR mice.

### 3.4 DEK knockout disrupts the activation of the RhoA pathway and inhibits Ezrin phosphorylation

**FIGURE 4:**
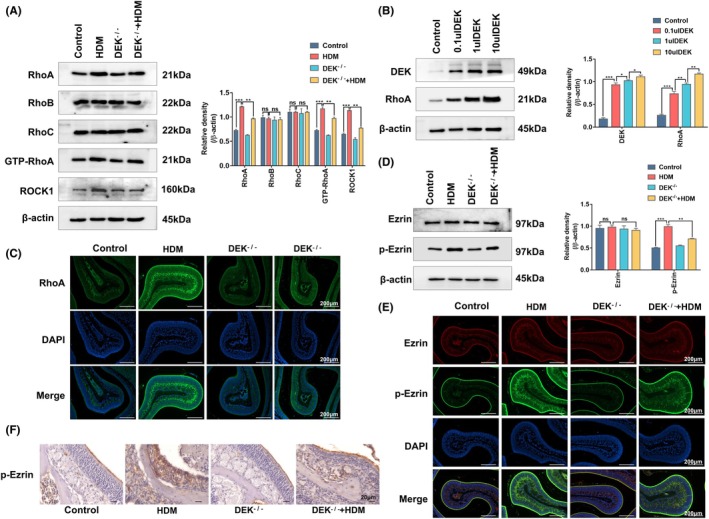
DEK knockout disrupts the activation of the RhoA pathway and inhibits Ezrin phosphorylation. (A, B, and D) Western blotting (WB) analysis of RhoA, RhoB, RhoC, GTP‐RhoA, ROCK1, and DEK protein levels in the nasal tissue mucosa of mice. (C and E) Immunofluorescence analysis of the expression of RhoA, Ezrin, and p‐Ezrin in the nasal sections of mice. (F) Immunohistochemistry analysis of the cellular p‐Ezrin expression in the nasal tissues of mice. Relative protein levels were calculated by dividing the protein density by that of β‐Actin, and the results are expressed as mean ± standard deviation. Magnification was 200×; ns, not significant; *p < 0.05, **p < 0.01, and ***p < 0.001.

Several studies have documented the close association of RhoA with allergic diseases,^23^, ^24^ whereas one study has indicated the regulatory effect of DEK on RhoA.^25^ The close relationship between Rho family proteins and Ezrin is well established, with RhoA frequently serving as an upstream pathway of Ezrin.^26^ Consequently, we conducted additional investigations to elucidate the association of DEK with RhoA, Ezrin, and AR. We first evaluated the expression of RhoA, RhoB, and RhoC before and after DEK knockout. As shown in Figure 4A, the RhoA expression significantly decreased following the DEK knockout. However, there was no significant change in the expression of RhoB or RhoC. Next, we introduced rmDEK at varying concentrations and found that RhoA increased in a dose‐dependent manner with increasing rmDEK concentration (Figure 4B). This suggests that DEK indeed exerts a regulatory effect on RhoA, and a positive correlation exists between the two. Subsequently, we evaluated the expression levels of RhoA and p‐Ezrin after the DEK knockout through immunofluorescence, and p‐Ezrin was significantly expressed in submucosa after HDM stimulation, whereas the expression decreased after DEK knockout (Figure 4C,E). At the same time, the expression levels of Ezrin and p‐Ezrin were detected again using WB (Figure 4D). The expression of p‐Ezrin was also detected using immunohistochemistry (Figure 4F). Based on these findings, it is evident that DEK knockout suppressed the activation of RhoA, consequently impacting the phosphorylation of its downstream pathway Ezrin.

### 3.5 The effect of DEK deficiency on apoptosis and mitochondrial fission in HNEpCs

**FIGURE 5:**
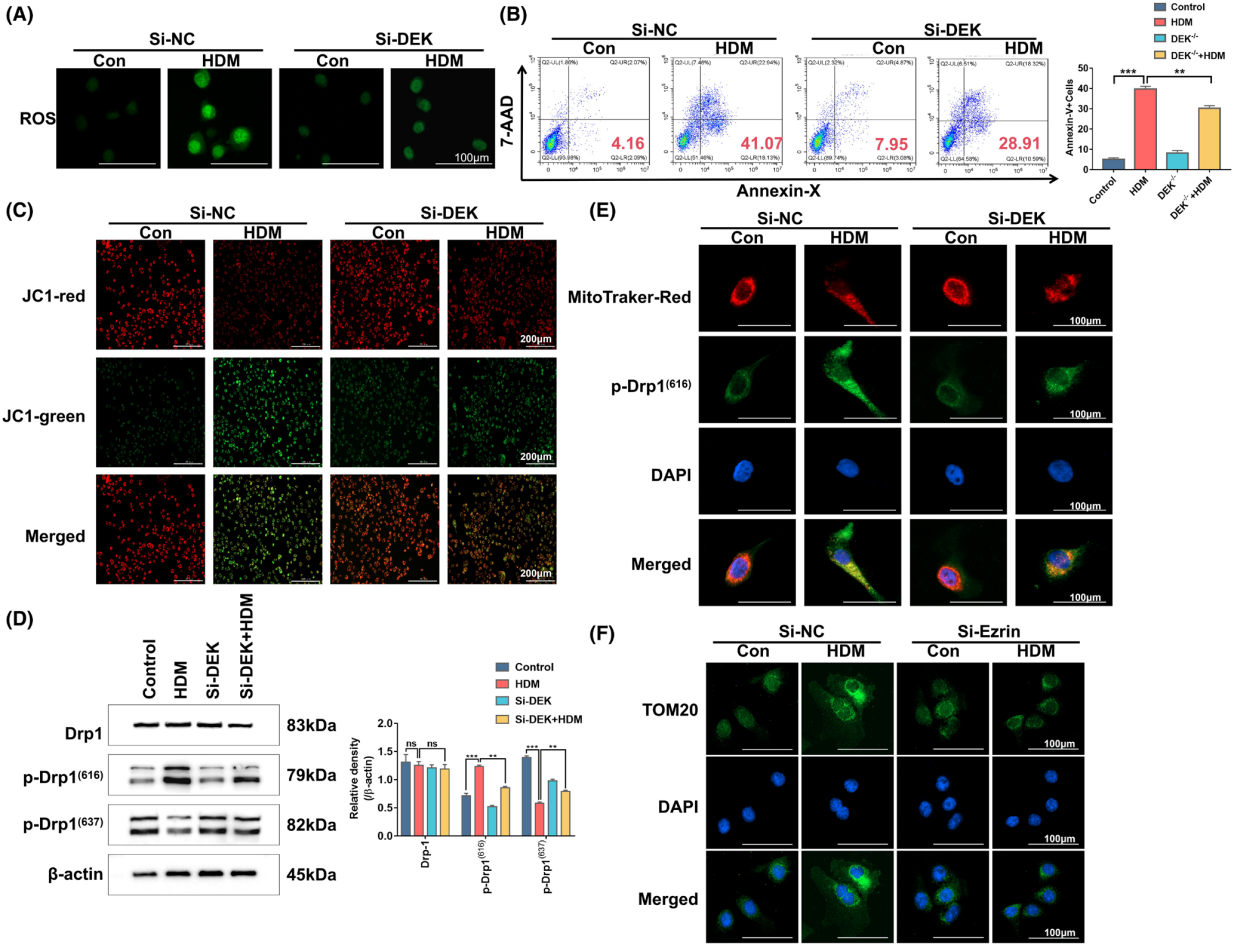
Si‐DEK alleviates apoptosis damage and mitochondrial fission in human nasal epithelial cells (HNEpCs). (A) Reactive oxygen species (ROS) generation in HNEPCs was measured with a DCFH‐DA probe. (B) Flow cytometry analysis of apoptosis was performed with 7‐AAD/Annexin‐V. (C) Mitochondrial membrane potential of HNEpCs after house dust mite (HDM) treatment was detected with JC‐1 dye. Scale bar = 200 μm. (D) Western blotting (WB) analysis of Drp1, p‐Drp1(616), and p‐Drp1(637) protein levels in the nasal tissue mucosa of mice. (E) Co‐localization of MitoTracker Red and p‐Drp1(616) in HNEPCs. (F) Tom20 assay was used to assess mitochondrial morphology in HNEPCs. Relative protein levels were calculated by dividing the density of the target proteins by that of β‐Actin, and the results are presented as mean ± standard deviation. ns, not significant; **p < 0.01 and ***p < 0.001.

Subsequently, we examined the effects of DEK deficiency on cell apoptosis and mitochondrial fission in vitro. ROS are inevitable by‐products of cellular metabolism, and their high levels directly or indirectly contribute to cellular signaling and induce apoptosis, which is a common pathogenic factor in diseases such as diabetes, atherosclerosis, and cancer.^27^ First, we utilized ROS to measure the overall level of cellular apoptosis, which revealed that the production of ROS significantly increased in the HDM‐stimulated cell group. Following si‐DEK treatment, the HDM‐stimulated group exhibited a decrease in ROS levels. Next, we used flow cytometry, TUNEL, and JC‐1 staining to more intuitively display the apoptosis level of HNEpCs. These findings collectively suggest that the reduction of DEK has alleviated apoptosis in HNEpCs (Figure 5A–C).

To investigate the regulation of mitochondrial fission by the downregulation of DEK, we first assessed the expression levels of Drp1 and its phosphorylated and dephosphorylated forms using WB. The results revealed no significant difference in the total expression of Drp1 among the four groups. However, the levels of phosphorylated Drp1 were markedly elevated in the HDM‐induced group and, to some extent, reduced after si‐DEK treatment. Conversely, the trend of dephosphorylation was observed to be the opposite. These findings suggest that knockdown of DEK can inhibit the phosphorylation of Drp1, thereby attenuating mitochondrial fission (Figure 5D). Subsequently, utilizing MitoTraker Red and Drp1 double staining, we observed an increase in phosphorylated Drp1 in the HDM‐treated model group, followed by a decrease in its expression after si‐DEK treatment (Figure 5E). Finally, we evaluated the morphological changes in mitochondria using Tom20 cell immunofluorescence. Relative to the HDM group, the morphology of HNEpCs mitochondria in the si‐DEK‐treated HDM group transitioned from fragmented or punctate to a reticular structure (Figure 5F). These results collectively suggest that the absence of DEK can reverse cell damage and mitochondrial fission.

### 3.6 si‐DEK disrupts the activation of the RhoA pathway and inhibits Ezrin phosphorylation

**FIGURE 6:**
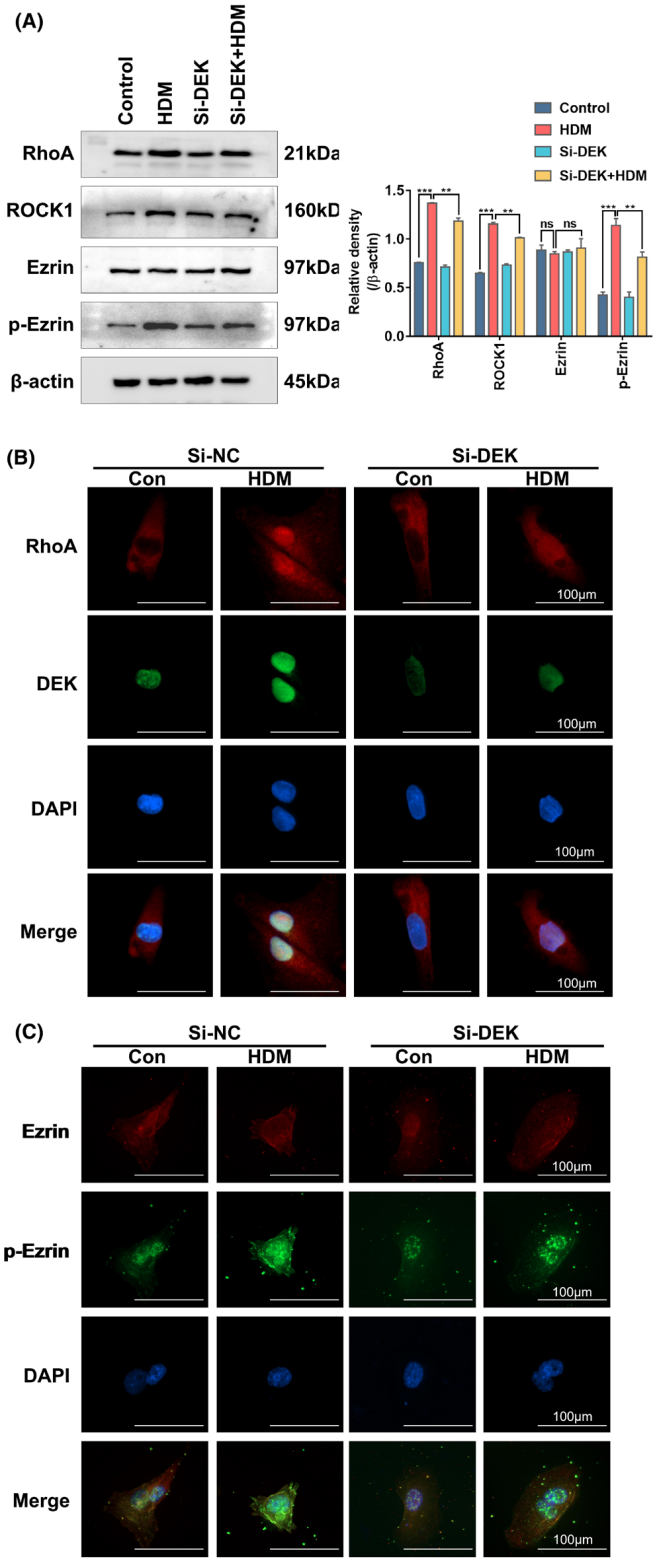
Si‐DEK disrupts the activation of the RhoA pathway and inhibits Ezrin phosphorylation. (A) Western blotting (WB) analysis of RhoA, ROCK1, Ezrin, and p‐Ezrin protein levels in the nasal tissue mucosa of mice. (B, C) Immunofluorescence analysis of the expression of RhoA, DEK, Ezrin, and p‐Ezrin in human nasal epithelial cells (HNEpCs). Scale bar = 100 μm. Relative protein levels were calculated by dividing the protein density by that of β‐Actin, and the results are presented as the mean ± standard deviation. ns, not significant; **p < 0.01 and ***p < 0.001.

In HNEpCs, we investigated the relationship between DEK, RhoA, and Ezrin. We initially exposed two cell groups to si‐DEK treatment for 24 h, whereas the other two groups received negative control treatment. Subsequently, one group from each treatment condition was subjected to the same dose of HDM for modeling. Western blot analysis revealed a significant downregulation of RhoA and p‐Ezrin expression in the si‐DEK‐treated model group compared to the untreated model group (Figure 6A). Similarly, the immunofluorescence showed similar results, along with the observation of RhoA translocating partially from the cytoplasm to the nucleus in its activated state during RhoA and DEK double staining (Figure 6B). What's interesting is that p‐Ezrin translocating from the cytoplasm to the cell membrane (Figure 6C). These findings are consistent with our previous mouse experiments, where DEK knockout or inhibition led to downregulation of the RhoA pathway activation and subsequent inhibition of Ezrin phosphorylation.

### 3.7 Ezrin can interact with Drp1 and mediate mitochondrial fission by regulating p‐Drp1^(616)^

**FIGURE 7:**
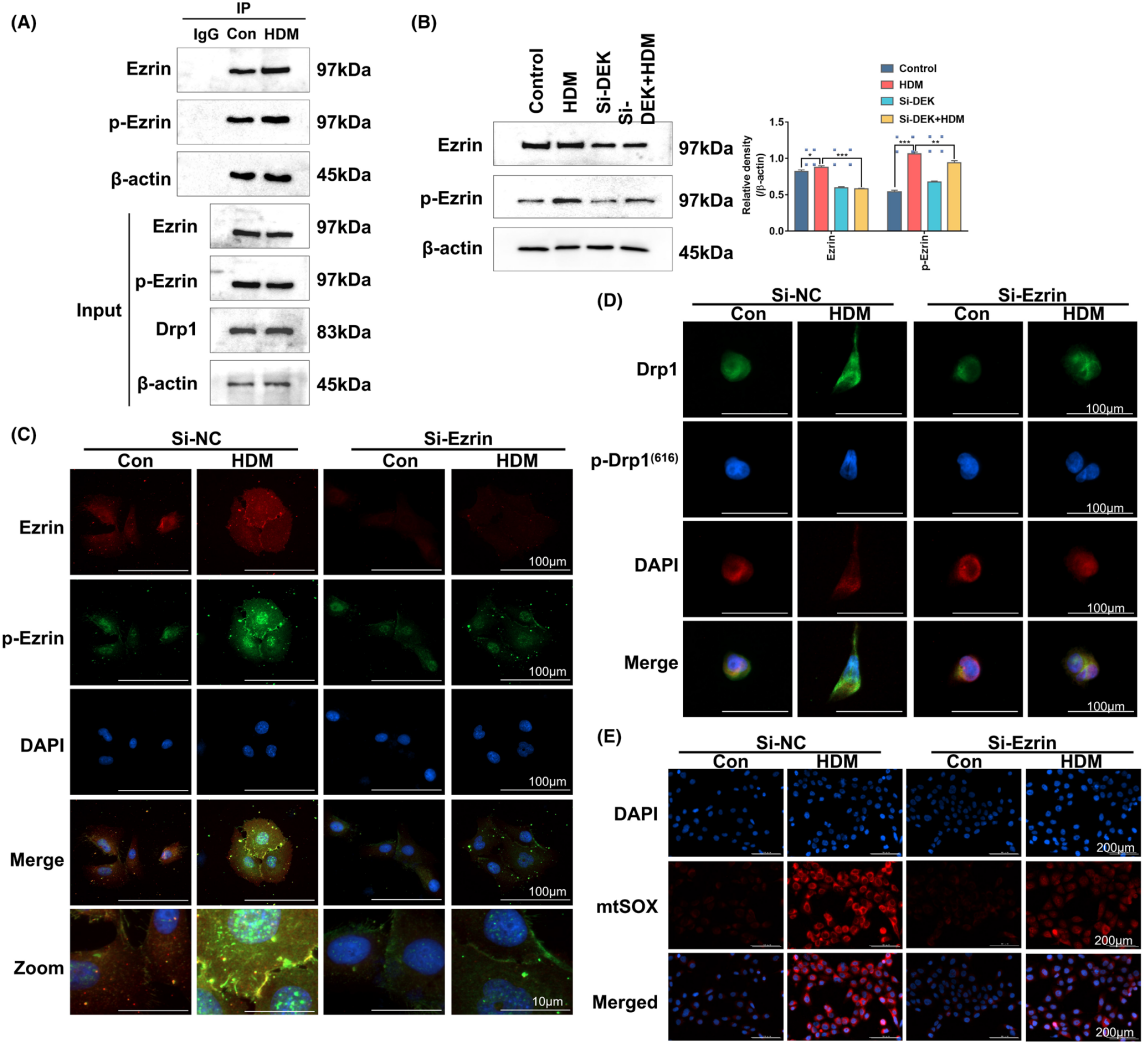
Ezrin can interact with Drp1, and after Si‐Ezrin, p‐Drp(616) decreases, and mitochondrial fission is alleviated. (A) Co‐IP analysis of the interaction between Drp1 and Ezrin. (B) Western blotting (WB) analysis of Ezrin and p‐Ezrin protein levels in the nasal tissue mucosa of mice. (C, D) Immunofluorescence analysis of the expression of Ezrin, p‐Ezrin, Drp1, and p‐Drp1(616) in human nasal epithelial cells (HNEpCs). (E) MitoTracker‐Red was used to observe mitochondrial morphology in HNEPCs. Scale bar = 100 μm. Relative protein levels were determined by dividing the density of these proteins by that of β‐Actin, and the results are expressed as the mean ± standard deviation. ns, not significant; *p < 0.05, **p < 0.01, and ***p < 0.001.

Based on previous results, we can confirm that DEK can mediate mitochondrial fission and affect the expression of Drp1, which is involved in regulating the pathway protein Ezrin and other pathway proteins. Therefore, we have made reasonable speculations about the reciprocal regulation between Drp1 and Ezrin. Therefore, we first explored the relationship between Ezrin, p‐Ezrin, and Drp1 by utilizing co‐IP. The results revealed that both Ezrin and p‐Ezrin could bind to Drp1, and the interaction was significantly enhanced after HDM stimulation, confirming that there is indeed an interaction between the two in HNEpCs (Figure 7A). To further determine the relationship between the Drp1 and Ezrin, we transfected HNEpCs with Ezrin‐specific short hairpin RNA (shRNA) (st‐h‐Ezr) and stimulated them with HDM. We observed the expression and localization of Ezrin and p‐Ezrin. St‐h‐Ezrin downregulates the expression of Ezrin and p‐Ezrin and inhibits their cytoplasmic transfer to the cell membrane (Figure 7B). Subsequently, we assessed the expression of Drp1 and its phosphorylated form (p‐Drp1^(616)^) in the Ezrin knockdown group by WB. The Ezrin knockdown model group showed a downregulation in the expression of p‐Drp1^(616)^ compared to the model group. These results were further validated through immunofluorescence (Figure 7C,D). Mitochondrial fission is typically associated with mitoSOX production, prompting us to assess its expression via cell fluorescence under Ezrin knockdown and HDM treatment. Cells treated with HDM exhibited a marked reduction in mitoSOX expression, as supported by the decreased mitoSOX expression under Ezrin knockdown and HDM treatment (Figure 7E), indirectly suggesting that Ezrin may regulate mitochondrial fission.

## 4 DISCUSSION

AR is an immunoreactive disease characterized by increased expression of specific IgE following stimulation of the nasal mucosa by allergens.^20^ However, the wide variety of allergens makes it difficult to identify inducible factors, leading to an unclear understanding of the pathophysiological process and the mechanisms underlying occurrence and development. Overexpression of eosinophils is a typical characteristic of AR morbidity and exacerbations. During the late‐phase allergic response in AR, there is the presence of inflammatory cells, such as eosinophils, basophils, and Th2 T lymphocytes.^28^ Group 2 innate lymphoid cells (ILC2s) are well known as key controllers of type 2 inflammation and are highly increasing in human airway type 2 inflammatory diseases, including AR, chronic sinusitis with nasal polyps, and asthma. In these diseases, ILC2‐mediated production of type 2 cytokines IL‐4, IL‐5, IL‐13, and others activates eosinophils, B cells, mast cells, macrophages, fibroblasts, and epithelial cells to initiate and amplify airway inflammation.^29^ Our current study demonstrates that exposure to HDM can induce overexpression of eosinophils. Furthermore, we found that DEK regulated and mediated the mitochondrial changes induced by AR through its effect on the RhoA/Ezrin signaling pathway. These findings suggest that inhibiting DEK and the RhoA/Ezrin signaling pathway could offer a novel therapeutic strategy for decreasing eosinophils and alleviating allergic symptoms in AR.

DEK, as a proto‐oncogene, plays a crucial role in cancer development.^30^ Additionally, there are studies that highlight the association between DEK and inflammation. Notably, DEK, a nuclear chromatin protein, acts as a chemical attractor inducing juvenile idiopathic arthritis.^31^ DEK is also important for the development of arthritis in mouse models, and the elimination or depletion of DEK can effectively relieve arthritis. It also significantly impairs neutrophilic extranuclear trap formation.^32^ Song et al. showed that DEK was overexpressed in asthmatic mice, and the inhibition of DEK could mitigate inflammatory cell infiltration and serum IgE level.^7^ Despite the known associations of DEK with inflammation and asthma, the role of DEK in AR remains unclear. Our study revealed the upregulation of DEK expression in the nasal mucosa and epithelial cells of mice with HDM‐induced AR. Knockout or knockdown of DEK led to alleviation of AR symptoms, reduction in inflammation‐related factors, and a significant decrease in eosinophil count in the nasal tissues and NALF. Furthermore, inhibition of apoptosis levels in nasal tissue sections and nasal mucosal proteins was observed, and alterations in the expression of mitochondrial dynamin Drp1 and its phosphorylated or dephosphorylated forms prompted further investigation. Therefore, the relationship between DEK and AR warrants additional investigation.

To investigate the role of DEK in AR, we conducted a literature search. Wang et al. reported that DEK deletion inhibited the RhoA/ROCK/MLC signaling pathway, thereby reducing cell migration in lung cancer cell lines.^6^ It has been demonstrated that RhoA and Ezrin signaling plays a role in inflammatory diseases. Zhou et al.^24^ demonstrated that RhoA's participation was necessary for the phosphorylation of Ezrin, which in turn caused endothelial cell hyperpermeability in PMVECs (pulmonary microvascular endothelial cells). Furthermore, RhoA, Ezrin, NHERF1, and actin form multiprotein complexes that are involved in reorganizing the actin skeleton network, thereby regulating the integrity and function of airway epithelia in cystic fibrosis.^33^ These findings suggest that DEK may modulate the activity of the RhoA/Ezrin signaling pathway, thereby mediating the development of AR. Although the involvement of DEK in AR pathogenesis remains unclear, our finding that HDM‐induced upregulation of DEK expression in the nasal mucosa of mice aligns with previous findings, indicating that DEK overexpression contributes to heightened allergic airway inflammation. Moreover, it is suggested that targeting DEK may alleviate AR. Consequently, we employed DEK knockout and wild‐type mice to investigate alterations in the RhoA/Ezrin signaling pathway following DEK knockout or inhibition. Our findings demonstrate that following DEK deletion, RhoA was activated into GTP‐RhoA, accompanied by a concurrent increase in total RhoA expression. Additionally, exogenous DEK stimulation resulted in a dose‐dependent change in total RhoA expression. Furthermore, as DEK is involved in RhoA activation, the phosphorylation level of Ezrin decreased following DEK knockout or inhibition, indicative of reduced RhoA activity.

Ezrin functions as a linker between the cytoskeleton and the cell membrane,^34^ establishing structural connections,^35^ which enhance the association between the cellular cortex and the plasma membrane and participate in various vital biological processes, including migration, proliferation, and endocytosis.^36^ Phosphorylation and intracellular localization of Ezrin are pivotal in regulating cell migration and biophysical properties, including membrane‐cortical junctions, cytoskeletal and nuclear organization, and mechanical support.^37^ Notably, our findings revealed specific expression of Ezrin and p‐Ezrin in the nasal tissue villi, with increased expression in squamous epithelial cells and submucosal cells following HDM induction. However, Ezrin has not been directly associated with AR. A study by Rebillard et al.^38^ illustrated that Ezrin underwent phosphorylation during GTPase‐RhoA activation after cisplatin stimulation, coinciding with an increase in apoptosis, which is consistent with our study. Apoptosis is frequently associated with an imbalance in mitochondrial fusion and division.^39^ In our study, we detected relevant indicators of apoptosis. We found that in the case of DEK knockout or inhibition, apoptosis‐related and mitochondria‐related indicators in nasal tissue and nasal mucosa cells were downregulated. Although the overall quantity of mitochondrial fission protein Drp1 remained relatively unchanged, the expression of phosphorylated Drp1 was partially inhibited. Notably, we showed that the activity of the RhoA/Ezrin pathway was also inhibited following DEK knockout or inhibition, prompting a study of the relationship between Ezrin and Drp1. Intriguingly, our Co‐IP findings revealed an interaction between Ezrin and Drp1. Following the transfection of cells with lentivirus to inhibit Ezrin activity, the phosphorylation of Drp1 was inhibited, implying the involvement of Ezrin in mitochondrial fission. The relationship between Ezrin and Drp1 is worthy of further study.

**FIGURE 8:**
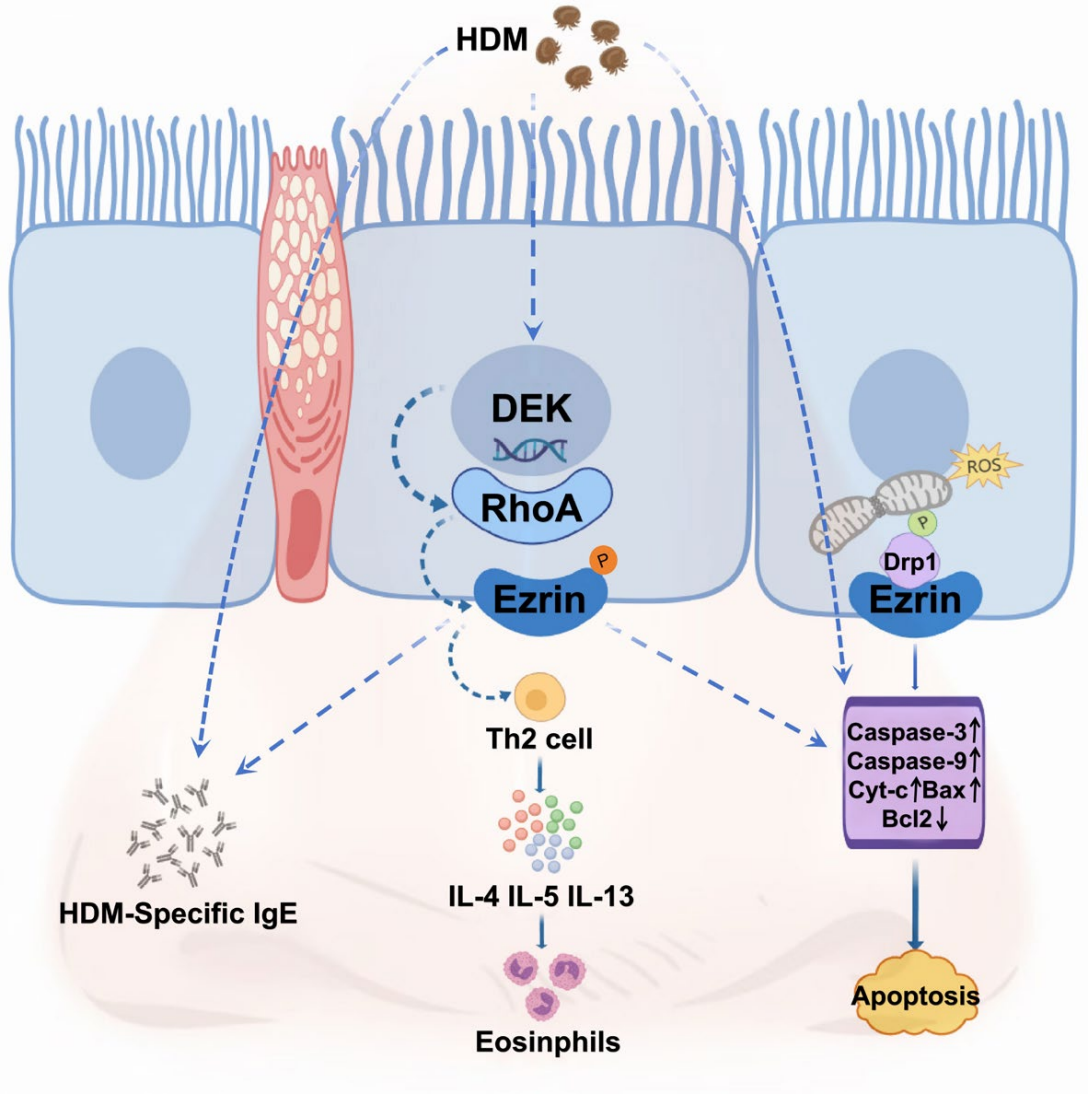
Schematic diagram of the mechanism of DEK regulating RhoA activation Ezrin. Upon house dust mite (HDM) stimulation, HDM‐specific immunoglobulin E (IgE) and inflammatory factors, such as interleukin 4 (IL‐4), IL‐5, IL‐13, and eosinophils, increased in the nasal mucosa. Furthermore, levels of mitochondrial fission and apoptosis were upregulated. Additionally, the increase in DEK activity activated RhoA and enhanced the phosphorylation of Ezrin, thus mediating the changes in the aforementioned rhinitis‐related indicators.

In summary, the results of our experiments indicated that HDM induced overexpression of DEK and an increase in the number of eosinophils in both nasal tissues and secretions. Subsequent investigations revealed that DEK knockdown or inhibition disrupted the RhoA/Ezrin pathway activation, leading to apoptosis and mitochondrial fission (Figure 8). Additionally, we identified the pathway protein Ezrin as a candidate for further investigation, as it was specifically expressed in chorionic, squamous epithelial, and submucosal cells of nasal tissue, and exhibited significant increases in response to allergen stimulation. Nevertheless, this study has some limitations. The representative significance of the proto‐oncogene DEK in AR remains uncertain, as there is insufficient literature to support a direct relationship between DEK and AR. An additional limitation is the difficulty in obtaining nasal mucosa samples from clinically diagnosed AR patients, which impedes the direct clinical relevance of our findings. Together, these studies highlight that DEK knockout or inhibition can mitigate the inflammatory response in AR mice, offering a novel approach to comprehensively elucidate the mechanism of AR.

## AUTHOR CONTRIBUTIONS

**Longzhu Dai:** Conceptualization; data curation; investigation; methodology; software; writing – original draft. **Yongde Jin:** Conceptualization; funding acquisition; investigation; methodology. **Jingmei Chai:** Data curation; visualization. **Jianing Yang:** Investigation; methodology. **Jiangang Wang:** Investigation; methodology. **Mu Chen:** Formal analysis; software. **Liangchang Li:** Data curation; visualization. **Chongyang Wang:** Conceptualization; funding acquisition; software. **Guanghai Yan:** Conceptualization; funding acquisition; project administration; supervision; writing – review and editing.

## FUNDING INFORMATION

This work was supported by National Natural Science Foundation of China (numbers: 82260007 and 82260218); the Department of Education of Jilin Province (JJKH20240698KJ); Natural Science Research Foundation of Jilin Province for Sciences and Technology (numbers: 20210101215JC, 20240602100RC, 20240404025ZP).

## CONFLICT OF INTEREST STATEMENT

All authors disclosed no conflict of interest that may directly or indirectly influence the content of the manuscript submitted.

## ETHICS STATEMENT

The experiment procedures were approved by the Laboratory Animal Ethics Committee Yanbian University (approved number: YD20231027002). The study adhered to the guidelines set by the committee.
